# Smallholders’ land access in Sub-Saharan Africa: A new landscape?

**DOI:** 10.1016/j.foodpol.2016.09.012

**Published:** 2017-02

**Authors:** Klaus Deininger, Sara Savastano, Fang Xia

**Affiliations:** a World Bank, United States; b University of International Business and Economics, Beijing, China

## Abstract

While scholars long recognized the importance of land markets as a key driver of rural non-farm development and transformation in rural areas, evidence on the extent of their operation and the nature of participants remains limited. We use household data from 6 countries to show that there is great potential for such markets to increase productivity and equalize factor ratios. While rental markets transfer land to land-poor and labor-rich producers, their operation and thus impact may be constrained by policy restrictions. Their functioning may also be constrained by ill-defined or insecure rights that may arise from failure to fully compensate existing rights in cases of expropriation, a failure to implement more broadly land policies or to do so in a gender sensitive manner. Methodological and substantive conclusions are derived.

## 1 Introduction

Scholars and policy makers alike have long recognized the importance of secure land tenure for sustainable land management, productivity-enhancing investment, operation of land markets that transfer land to its best and most productive use, and eventually of access to credit markets by using land as a collateral (Besley and Ghatak, 2010). Yet, while this has given rise to an array of interventions to formalize land rights in virtually all of the world’s regions, Africa remains in many ways an exception. A key reason is that in Africa, customary institutions traditionally provided levels of tenure security high enough to encourage investment (Bruce and Migot-Adholla, 1994). Given the continent’s traditional features including relative land abundance and producers’ use of rather simple un-mechanized technology with reliance on family labor, it is generally believed there is little scope for large investment that would require access to credit. This would imply limited scope for productivity differences across producers and consequently for land (rental) markets to improve productivity. Together with the high cost of formalization efforts, this has led to a widely held view that, in the African context, efforts to increase tenure security will be superfluous (Pinckney and Kimuyu, 1994), not sustainable (Atwood, 1990) or may, if they give rise to appropriation of land by well-connected individuals, even be harmful (Easterly, 2008).

Recently, studies started documenting that reality may diverge from this view in a number of respects. First, even without accounting for the recent demand for land acquisition by large farms in Africa (Deininger and Byerlee, 2011), productivity among smallholders may vary considerably even in non-mechanized settings (Restuccia and Santaeulalia-Llopis, 2015). Second, customary systems may be biased against women, in particular restricting their ability to inherit land and thus their bargaining power within the household. Also, as land becomes more scarce, traditional institutions may come under stress and be no longer able to ensure equitable land access. This is linked to increasing numbers of land disputes and possibly traditional chiefs transferring land for private gain rather than community benefit. Third, growth of the rural non-farm economy, rural-urban migration, and diversification of income sources increase the scope for efficiency-enhancing transactions as dispersion of skill increases, investment becomes more profitable, and land from those joining the non-agricultural can be leased to those specializing in agriculture. In such situations, rental markets can provide advantages by equalizing factor endowments and allowing gains from specialization (Holden et al., 2008, Jin and Jayne, 2013). If, as in commonly found, factor markets are imperfect (Dillon and Barrett, 2017) but production is labor- intensive with an inverse relationship between farm size and productivity, this will increase productivity. All of this implies that, in countries undergoing rapid economic change, regular and reliable data will be important to inform policy not only to indicate if reality still corresponds to common wisdom but also improve understanding of drivers and potential obstacles to structural transformation and growth of the rural non-farm economy.

We draw on large representative cross-sectional household data from 6 countries (Ethiopia, Malawi, Niger, Nigeria, Tanzania, and Uganda) collected under the auspices of the LSMS-ISA program between 2010 and 2012.^1^ In addition to providing updated and consistent information on differences across households in terms of endowments and productivity, we assess the extent to which rental markets help bring about the advantages expected from them. Variables available from the surveys, together with general information on the institutional environment prevalent in these countries, are used to identify determinants of land market functioning at a descriptive level. We estimate a reduced form model of land rental and sales market participation that makes use of the geo-referenced nature of the data at hand. In addition to affirming the scope for sizeable gains in equity and potentially efficiency from better functioning of land rental markets, we go beyond the existing literature, and we formulate: (i) hypotheses regarding obstacles to land market functioning that warrant discussion with policy makers and should be subjected to further empirical tests, and (ii) we suggest variables to include in future surveys that could inform such debates.

Main findings are as follows. First, we show that, contrary to expectations about limited potential of land rental, in the countries studied here, large differences in land endowments and productivity create potential for land market to equalize endowments and contribute to higher levels of productivity. Second, we find that land rental markets improve equity by promoting land access to those with limited land endowments. Finally, while a detailed examination of the impact of institutional factors is beyond the scope of this paper, our findings suggest that rental market performance seems lower where land rental is explicitly or implicitly outlawed, the threat of uncompensated expropriation increases tenure insecurity, and policies to document existing land rights, especially by women, are not implemented or out of reach for the majority of land holders. In addition to suggesting that there may be a need to revise traditionally held views on African agriculture and land markets, or at least to differentiate them in line with rapid changes of land scarcity, this also implies that greater attention to these issues in the policy and institutional dialogues and in future data collection efforts will be warranted.

The paper is structured as follows. Section two discusses conceptual background and literature regarding the role of land markets, their evolution over time, institutional pre-conditions for effective functioning, and welfare- and productivity impacts as well as institutional arrangements in study countries. Section three uses LSMS-ISA data to descriptively explore levels of documentation and productivity, as well as incidence and determinants of land rental market operation in the six countries. Section four explores determinants of participation in land rental and sales markets econometrically. Section five summarizes results and initial policy recommendations.

## 2 Background and justification

To contextualize issues, this section reviews the conceptual background and literature and the way in which they link to the context encountered in each of the study countries. Based on the notion that secure rights are required to provide the basis for their operation, land rental markets have been shown to be efficiency- and welfare enhancing all over the globe. While their potential in Africa is often assumed to be more limited, recent studies point towards high levels of variation across countries and show that this picture may be changing slowly in response to secular trends. Yet, a number of factors including a perceived threat of expropriation, women’s or other groups’ inability to obtain secure long-term right to the land they use, partial or ad-hoc implementation of certification program, or restrictions on transferability imposed to maintain rural equality or stem urban migration may in practice pose obstacles to their operation.

### 2.1 Conceptual framework

While Africa has traditionally been described as land abundant, the continent is characterized by enormous heterogeneity,^2^ much of the land that could potentially be available for expansion is concentrated in few countries (Deininger and Byerlee, 2012), often with poor fertility or infrastructure access (Chamberlin et al., 2014). Technology-induced limits on farmers’ ability to expand cultivated area and demand for African land from outsiders (Arezki et al., 2013, Schoneveld, 2014) imply that in many settings farm households have to adjust to higher levels of land scarcity, via investment and intensification of production or greater reliance on off-farm income, possibly alongside greater land rental market participation (Headey and Jayne, 2014). Continued high levels of population growth also point towards increasing land scarcity, as documented in Mali (Guirkinger and Platteau, 2014) or Ethiopia (Headey et al., 2014). All these factors suggest that economic development and structural transformation involve specialization and a shift of a part of the labor force out of the agricultural sector that create heterogeneity in the population, and increase the scope for efficiency-enhancing land transfers.

Markets for land sale or rental are largely absent in relatively land-abundant settings where the binding constraint is labor rather than land, and in subsistence economies where land is rather equally distributed, the skill-intensity of agricultural cultivation is low, and the availability of non-farm opportunities limited. These processes, together with economic diversification and growth in the non-farm sector, increase the scope for efficiency-enhancing land transfers beyond immediate kin and for periods that are likely to be longer than just one season. The literature documents that in this case, institutions, especially formal documentation of ownership to encourage long-term transfers extending beyond immediate family, can increase land use productivity and foster economic development and diversification (Badiane et al., 2012). Land markets will operate more smoothly if clear information on land ownership is available at low cost and social norms ensure that a landlord – who temporarily transfers land through rental -, does not risk the loss of this asset. This has historically provided the justification for public registries. Ill-defined or insecure property rights, including a failure of public institutions to respect and compensate existing rights in case of expropriation for public purpose, may put productivity-enhancing transactions out of reach for all or part of the population. Even if rights are clear and respected, inefficient institutions that make registering transactions costly and cumbersome will reduce the scope for land market transactions, e.g. by creating barriers to market participation (Sitko and Jayne, 2014). This can either undermine the ability to reap associated gains in terms of productivity and financial market development or drive such transactions into informality.

Empirical studies support this: Historically, reforms in Russia increased the scope for land leasing and promoted migration by easing financial constraints and decreasing opportunity costs (Chernina et al., 2014). More recently, a randomized roll-out of land registration in Rwanda helped to significantly improve land rental market operation and productivity (Ali et al., 2015b), providing a basis for an incipient mortgage market secured against agricultural land (Ali et al., 2016). Land certification in Ethiopia had positive impacts on investment (Holden et al., 2009) and land market participation (Deininger et al., 2011a). In India, improving producers’ access to information by computerizing land records helped to increase the number of registered transactions and credit access in urban but not rural areas (Deininger and Goyal, 2012). Under Mexico’s 1993–2006 land certification program, households obtaining certificates were 28% more likely to have a migrant member but there was no effect on cultivated area due to markets helping to consolidate farm units (de Janvry et al., 2015). In fact, clarification of inheritance and providing options for leasing out is estimated to have increased eligible households’ likelihood of having one or more members abroad by 12%, explaining 26% of the 1994–1997 increase in the number of migrants from the entire *ejido* sector (Valsecchi, 2014). In China, where sales are not allowed, issuance of certificates helped reduce the risk of leased out land being taken back and redistributed (Mullan et al., 2011), created institutional preconditions for households to take on non-agricultural activities (Deininger et al., 2014), and increase efficiency (Deininger and Jin, 2009).

As leasing does not require changes in land ownership, land owners, even if they give up self-cultivation, will not have to forsake benefits from owning land, including the ability to use land as collateral for loans to start non-agricultural enterprises (Deininger and Feder, 2001). To the contrary, it provides access to regular income streams in the form of rents (Deininger, 2003) which, if competitive markets help transfer land to more productive producers, will be higher than what owners could obtain from self-cultivation (Deininger et al., 2011). Households who participate in off-farm markets will be more likely to supply land to the rental market (Kung, 2002) and restrictions on land rental may limit the scope for off-farm development as in Ethiopia (Deininger et al., 2003a). Equalization of land access through rental markets is also observed in Kenya (Jin and Jayne, 2013), Uganda (Baland et al., 2003), Ethiopia (Deininger and Jin, 2006, Teklu and Lemi, 2004), Eastern Europe (Swinnen and Rozelle, 2009), and India (Deininger et al., 2008b).

Land sales may allow households who want to move into the non-agricultural economy to mobilize the equity that will help them to exploit profitable economic opportunities but they are less liquid and may be affected by credit and other factor market imperfections. Indeed, asymmetric information and risk have long been shown to lead to credit rationing in equilibrium and the use of collateral as one way of reducing such credit rationing (Stiglitz and Weiss, 1981). Its immobility and relative indestructibility make land an ideal collateral. However, banks will use it for this purpose on a large scale only if they have access to low-cost means to make reliable inferences on ownership and the absence of other encumbrances for any given plot of land. Such information is normally provided by land registries. Compared to rental, land purchases will require more resources and purchase decisions will also be affected not only by returns from productive use of land but also by expected future movements of land prices compared to those of other assets. Moreover, lack of insurance and highly covariate risk can cause price fluctuations whereby agricultural land is cheap in times of crisis (e.g. drought) when farmers have to resort to distress sales to cover their subsistence and very expensive in years with good harvests so that land sales markets may be less equity-enhancing than those for rental and can even increase inequality (Zimmerman and Carter, 2003).

### 2.2 Institutional background

The countries in our sample vary not only in terms of relative factor endowments but also in their institutional framework. One common issue is that as innovative legislation to document rights was often made without assessment of associated cost or underlying demands, implementation often faces considerable challenges. Also, in many contexts, implicit or explicit restrictions on rights may undermine the scope for effective use. Three issues often encountered are (i) explicit restrictions on land rental markets including the risk of land loss if land is leased out and thus perceived as not needed or not used effectively; (ii) neglect of existing rights in case of expropriation by providing compensation only for improvements or well below market values, often combined with a wide interpretation of state powers of eminent domain well beyond the narrow provision of public goods; and (iii) limited documentation of rights or ability to register them, an issue that would particularly discourage longer-term transactions.

Ethiopia’s highlands, where land is very scarce, implemented a participatory, low cost, and gender-balanced effort to provide documentation of rights to users. This reduced transaction cost and made land transfers easier, in a context where land sales are not allowed and, in many regions, leasing of a household’s entire holding is prohibited to prevent rural-urban migration.^3^ Also, if land is converted from rural to urban use, failure to recognize rural holders’ rights is likely to cause tenure insecurity at the urban fringe (Adam, 2014). A similar issue is confronted in Nigeria where a tenure regime based on state ownership established under the 1978 Land Use Act severely restricts the scope for having eliminated any private land ownership and creating far-reaching scope for state interference. Nigeria is characterized by a regime that creates high levels of tenure insecurity, undermines investment, and drives transactions underground (Adenyi, 2011). In Niger, land tenure has long been shown to affect investment (Gavian and Fafchamps, 1996) but enlightened legislation to facilitate integrated management of natural resources (Wabnitz, 2009) has little impact due to limited implementation (Cotula, 2008). Malawi is not only land scarce but also has a dualistic tenure system where estates, many underutilized or encroached, exist side by side with customary institutions land. A Land Bill has been in Parliament for over a decade (Peters and Kambewa, 2007). Inheritance can follow patriarchal and matriarchal lines. While women’s land access alone may not increase productivity (Bhaumik et al., 2013), land inheritance has been identified as a critical determinant of women’s empowerment including their access to divorce (Telalagic, 2012). In Tanzania, the 1999 Village Land Act vests all rural land in village councils who are mandated to elaborate participatory land use plans as pre-condition for issuance of certificates of customary ownership. Beyond implementation challenges (Pedersen, 2012), the requirement of having any transfer involving individuals from outside the village approved at different levels greatly increases the transaction costs for land transfers and reduces predictability (Deininger et al., 2011b). In Uganda, overlap of rights between tenants and landlords on *mailo* lands that reduces productivity and investment (Deininger and Ali, 2007) also makes transfers more difficult. With complex processes and top-heavy structures, the costs of the country’s 1998 land law were estimated to be prohibitive (Hunt, 2004) and implementation has not yet moved beyond the pilot stage.

## 3 Data and descriptive statistics

Household survey data allow us to explore determinants and impacts of the operation of land markets, the extent to which such markets contribute to greater productivity, and barriers to their functioning. We briefly characterize coverage of surveys available and use these to describe the nature of the rural economy overall, estimated levels of participation on both sides of land rental and sales markets, and characteristics of participants in both types of transactions.

### 3.1 Data and sample description

We use data from LSMS-ISA surveys in Ethiopia, Malawi, Niger, Nigeria, Tanzania, and Uganda. All of these are based on large, representative multi-purpose household surveys with detailed information on agricultural production that are representative either for the entire country or for rural areas within a country. In addition to having been designed as panels so as to allow controlling for household-fixed effects, these data have three advantages. First, they aim to consistently use GPS to measure plot size, to reduce the measurement error inherent to farmers’ estimates (Kilic et al., 2013) that can have far-reaching impacts for estimates of outcome variables such as yield (Carletto et al., 2013). Second, while phrasing of questions differs somewhat across surveys, they allow retrieval of information on the gender of the plot manager or owner.^4^ This allows to appreciation dimensions of asset ownership or control by gender and potential implications for efficiency of resource use (Aguilar et al., 2014). Finally, all surveys provide GPS coordinates of at least the homestead so as to be able to link to infrastructure access and other physio-geographic data including agro-ecological potential (Fischer et al., 2002) that may have an important impact on production, prices for input and output, and the ease with which non-farm opportunities can be accessed.

A limitation of our data is that samples are household-rather than area-based and in some cases limited to rural areas. Land held by legal entities or corporations, or in cases of rural samples by urban dwellers, will thus not be captured. This may affect estimates of the farm size distribution in relatively land-abundant countries where these groups can account for sizeable land holdings. In Zambia for example farms operating between 5 and 100 ha of land, most owned by urban dwellers or companies, have been found to control more land than the country’s entire smallholder sector (Sitko and Jayne, 2014). The importance of this gap will vary across countries depending on the legal framework and the ability to actually implement it, an issue that on which further research would be very desirable. For Ethiopia, a commercial farm survey is available which puts the total area held by operational commercial farms at 1.55 million hectares (Ali et al., 2015b), compared to 9.2 million hectares cultivated by smallholders so that our data would account for some 85% of the land used for agriculture in the country.^5^

### 3.2 Country context

Descriptive statistics for the surveys in our sample provide a number of insights (Table 1), pointing in particular to a wide distribution of land ownership, large productivity gaps across producers, low levels of formal documentation of land ownership, low frequency of land sales *versus* rentals, and sizable variation of land market activity across countries, partly in response to land scarcity and possibly also to institutional factors. The surveys reveal marked differences across countries in the shares of illiterate heads (from 66% in Ethiopia to 16% in Uganda) and mean household income (from USD 664 to 1396 in Niger though per capita income is more equally distributed (from USD 323 in Nigeria to USD 205 in Malawi). They highlight that smallholders rely on diverse and dynamic livelihood strategies (Davis et al., 2017): while engaged mainly in agriculture, the majority of households complement agricultural income with resources from other areas. In fact, except for Nigeria where 70% of household income is from crops, the mean contribution of crops and income from crop production is less than 50%, with agricultural wages (from 1% in Nigeria to 15% in Malawi), self-employment (from 6% in Malawi to 24% in Niger), and transfers (from 1% in Nigeria to 8% in Malawi) making up the rest. Migration is also widespread with 17% of households in Uganda as well as 13% in Malawi and 9% in Tanzania having at least one member who migrated to their current location of residence in search of work or land. Conversely, 21% in Niger and 8% in Uganda have a member who migrated out for economic reasons (search for land or work).

While countries in our sample differ from each other in terms of relative land scarcity (Jayne et al., 2010), operated area per adult household member in smallholder farming is often limited by technology (Binswanger, 1986) and thus less variable across countries. Indeed, for all of countries except Niger, operated area per adult is less than one ha. With 3.02 plots per household, the level of fragmentation is highest in Ethiopia and lowest in Malawi (1.74 plots per household) with many of those with little or no land engaging in wage employment.

For all countries except Ethiopia we can compute the value of output as well as profits per ha.^6^ Doing so points towards variations in levels of land and labor productivity. With gross per-hectare income from crops between USD 267 in Malawi and USD 545 in Uganda,^7^ net income per hectare ranges from some USD 164 in Malawi to USD 289 in Tanzania. Combining this with cultivated area and labor by family workers allows us to obtain a range of net income per day of family labor between USD 2.21 for Malawi and USD 1.19 for Uganda. As this figure includes returns to land and other fixed assets, it provides an upper bound estimate of the reservation wage, at which the average individual would likely be indifferent between working in own agriculture and taking on non-agricultural wage work or engaging in non-agricultural self-employment.

With between 93% (in Malawi) and 62% (in Uganda) of households having acquired land in this way, inheritance or grant by traditional authorities or the extended family remains the main way for accessing land. Levels of formal or informal documentation of land ownership remain low throughout. Except for Ethiopia where 38% of households indicated to have a formal document that covers close to 50% of owned area due to the country’s participatory low-cost land certification program (Deininger et al., 2008a), the share of households with any type of document is below 20% everywhere else with the share of area with formal document (certificate of title or customary ownership) reaching 12% in Uganda, mostly *mailo* titles that were issued by the colonial administration.^8^ It suggests that, although legislation to issue documents to customary land exists in many countries and households’ demand for formal documentation in countries such as Tanzania is high (Ali, 2016), implementation gaps may prevent these from having their full impact.

Plot managers’ gender varies markedly across countries; male managers are in charge of some 80% of area in Ethiopia and Nigeria, 71% in Malawi, and 53% in Niger but only 27% and 9% in Tanzania and Uganda where some 45% and 52%, respectively, are under joint management and 24% of 38% managed by females on their own. While only 7% or 30% of households report the owner’s gender in Nigeria and Ethiopia, almost all do so in Uganda and some 80% in rest of the countries. Female ownership is most widespread in Malawi with 45%, followed by Uganda (28%), Tanzania (22%), and Ethiopia (18%).

Summarizing geo-spatial variables highlight differences of population density between the enumeration areas (EAs) selected in the survey ranging from less than 100 persons/km^2^ in Niger (49) and Tanzania (65), 100–200 in Ethiopia (142) and Malawi (175) and more than 200 in Nigeria (207) and Uganda (268). Comparing these to the distance to the next road (from 8 and 9 km in Uganda and Malawi to 17 and 22 km in Nigeria and Tanzania) shows that access to infrastructure is only weakly correlated with population density and a similar conclusion emerges with respect to light intensity and urban gravity.

Information on land rental and land sales in Table 2 illustrates that, with 21% and 19%, respectively, the share of households renting in land is highest in Ethiopia and Uganda and, with 6% or 7%, lowest in Tanzania and Niger. Malawi and Nigeria are in between with a market participation of 10% or 11%. By comparison, the share of households who, in the survey, report to rent out land is much lower, being highest in Ethiopia with 5%, followed by Niger (1.6%), Tanzania (1.1%), and less than 1% in Uganda, Malawi, and Nigeria. To explore if, as theoretically possible, this discrepancy may be due to landlords leasing out large tracts of land to multiple tenants, we also present the total area which, according to the survey has been leased in or out using survey weights (which refer to population). Part of the under-estimation of leased out land may be due to the fact that, at least in some countries, landlords may move to urban areas and thus either not be included in the sample or not respond to questions on leasing out land that may be included in the agricultural section of the survey (Deininger and Jin, 2008).^9^ Yet, differences in the share of leased in area not accounted for on the other side are pronounced; while in Tanzania and Niger about 50% and 30% of the area leased in is also reported to have been leased out by a landlord, this figure is only 14% for Ethiopia and 6%, 4%, and 1%, respectively for Uganda, Malawi, and Nigeria.^10^ While the surveys allow inferences on determinants of renting out, getting a fuller picture of land market functioning will require information on both sides of land markets.^11^

Data on land acquisition by the current household to provide information on land purchases is available for Uganda and Niger where 10% and 4% of households, respectively, indicated that they bought land during the past 5 years. While high levels of land rental as well as sales market activity in Uganda have long been noted (Baland et al., 2007), though sales market activity is still below what is observed in Rwanda (Ali et al.), detailed analysis is possible only for Uganda and Niger. Plots of the density of owned or operated land point towards a skewed distribution virtually everywhere, with a vast number of very small plots but a long tail of some larger ones. To illustrate distributional aspects, Table 3 tabulates mean owned and operated area by quartile of the land ownership distribution. Mean owned areas for the top quartile of 1.48 ha in Malawi, 1.88 ha in Nigeria, 2.53 ha in Ethiopia, 3.54 ha in Uganda, 6.97 ha in Tanzania, and 11.6 ha in Niger highlight differences in relative land scarcity. Also, the redistributive effect of land rental is evident from the fact that in many countries operated area by the bottom quartile is double what is owned whereas everywhere the area operated by the largest land owners is less or equal what they own.

### 3.3 Land rental market participation

A key driver of the operation of land markets are differences in land ownership and productivity. Data on these variables can thus help assess the potential for land market operation. Marked differences between the bottom and top quartiles in both for most countries in our sample as illustrated in Table 3 points towards considerable scope for equalizing factor endowments and productivity through market operation. While differences between the top and the bottom quartile average 20 for land ownership (panel A) and even more for productivity (panel B) possibly due to outliers, inter-quartile ranges are from 1.64 for Malawi to 2.96 for Nigeria in terms of land ownership and 2.26 to 3.44 for these countries in terms of productivity. Also mean monetary output per ha is higher for smaller farms (panel C). Comparing to the operational distribution of land in panel D suggests that markets contribute to narrowing these gaps -the top to bottom quartile ratio narrows to about 10. Still, with the mean inter-quartile range remaining above 2, in all of the countries concerned, land markets fall short of achieving their potential in terms of factor equalization and are more effective to cut the extremes of the distribution.

To descriptively explore determinants of land market performance, Table 4 displays statistics separately for households who did and did not participate in land rental markets.^12^ With a large part of those renting in land being landless (74% in Nigeria, 54% in Malawi, 35% in Tanzania, and 25 and 24% in Niger and Uganda, respectively), land rental allows the land poor to access land. In Ethiopia and Malawi, lessees have more labor than non lessees and have higher levels of human capital. While this suggests that, beyond improving equity, land rental could also allow more productive land use. Lessees’ yields are, however, significantly higher than those of non-lessees’ only in Uganda.

Younger household heads are more likely to rent in land to expand the size of their agricultural holding although with an average head age of beyond 40 -from 40 in Malawi to 49 in Nigeria- intergenerational mobility and youth access to land may still be an issue that warrants attention. While the share of those without any education is lower among those renting in than the rest everywhere, possibly pointing towards a minimum level of functional literacy, participation in rental markets does not seem to be associated with high levels of formal education except in Malawi where educated wealthy households seem to access land through rental markets. Households headed by females are less likely to rent in land in Ethiopia, Malawi and Niger, something that may partly be a result of their lower overall labor endowment. To the extent that information on the value of assets is available, we note that lessees in Niger and Nigeria but not in Uganda hold more agricultural assets. Interestingly, land is transferred to wealthier households ($2741 vs. $1604 value of durable goods but not agricultural assets) in Malawi. In Malawi and Uganda and to a lesser degree in Tanzania, households with in-migrants are more likely to lease in land while in Niger those with out-migrants are more likely to rent in.

Non-farm diversification seems a key driver of land rental activity. In Malawi, Nigeria, and Uganda, land markets are more active in the enumeration areas with greater non-agricultural employment although levels of land market activity do not seem to vary with local population. Information on tenants’/landlords’ non-agricultural incomes would allow to ascertain if/how land markets contribute to structural transformation by either allowing interested individuals to take up non-agricultural employment without losing the safety net function implied by land ownership and making effective use of their land (Deininger et al., 2011c) or by alleviating credit constraints.

### 3.4 Land sales market participation

Data on land sales are available only for two countries -Uganda and Niger. While including questions on land acquisition in samples of agricultural cultivators is important, land may increasingly also be acquired by firms that may not be visible in household surveys (Headey and Jayne, 2014, Schoneveld, 2014). Analysis of such transactions can be of great policy relevance (Sitko and Jayne, 2014) and will be important especially in land-abundant countries where, in the context of the post-2008 food price spike (Deininger and Byerlee, 2011), magnitudes involved are likely to be large and thus will affect the agricultural sector more broadly (Jayne et al., 2014). Use of data on land transfers from registries as a frame is one option to cross-check and complement data from household samples that would also allow obtaining ‘objective’ land prices.^13^

Descriptive statistics for households who did or did not purchase land over the last 5 years in Table 5 for Uganda and Niger suggest that in Uganda households with in-migrants are more likely to have purchased land than remained in autarky or sold. Sales markets had a mildly redistributive effect and transferred land to those with lower land endowments.^14^ At the same time, those who purchased land expanded existing holdings, are better educated, have significantly higher income and assets, and apply slightly more inputs than those in autarky. Infrastructure access seems to matter less for land purchases than for rentals, something that could be due to a number of reasons (e.g. buyers having access to transport).

## 4 Econometric exploration of determinants of land rental and sales participation

As, for most countries, information on leasing out in our data is too thin for meaningful estimates,^15^ we assess determinants of land rental market participation by estimating a probit equation of the form:

(1) $$R_{\mathrm{ij}}=\alpha +\beta X_{\mathrm{ij}}+\gamma Z_{j}+\varepsilon_{\mathrm{ij}}$$

where *i* and *j* index households and enumeration areas, respectively, *R_ij_* is an indicator variable for households renting in land, *X_ij_* is a vector of household characteristics, *Z_j_* is a vector of location-specific variables,^16^ and *β* and *γ* are vectors of parameters.

Results, reported in Table 6 for specifications with geo-variables (density, distance to road, non-agricultural income shares) allow to complement and substantiate the conclusions from descriptive statistics. The main conclusion is that, while land rental can play an important role to equalize land endowments and land/labor ratios, allow more efficient producers access to access land, and contribute to movement of labor out of agriculture, the extent to which it does so varies widely across countries in ways that partly are linked to institutional factors. Subject to data limitations, analysis suggests that, while land rental markets fail to fully equalize factor ratios, they contribute to structural transformation by helping to transfer land to land-poor and relatively labor-rich households.

A finding that is very consistent across countries is that land markets contribute to equalization of land endowments. In all countries, lower land endowments increase the propensity of land market participation, with estimated effects largest in Malawi, Nigeria, and Uganda where overall land pressure is high and rather modest in Tanzania, Niger, and Ethiopia. Evidence of labor market equalization is more ambiguous with positive signs on the size of the adult labor force aged 14–60 only in Ethiopia, Malawi, Niger and Uganda but not in Nigeria and Tanzania. The negative coefficient in the rent-out equation for Ethiopia points towards some equalization there as well (reported in the appendix table). With the exception of Ethiopia, head’s age is not significant. At the same time, regressions point towards females being less likely to rent in land in many countries, even if differences in other factor endowments are accounted for, with effects most pronounced in Ethiopia (in line with that of the rent-out equation reported in the appendix table), followed by Nigeria, Niger and Malawi. Coefficients on assets are significant only in Malawi and those on yields (reported in the appendix table) in Malawi and (weakly) in Niger.^17^

In Malawi, households with members who migrated in are significantly more likely to lease in- or marginally significant coefficients in Uganda. Geo-variables add interesting insights; while higher population density is associated with higher levels of rental activity in Malawi and Niger, the relationship is insignificant in Uganda and Tanzania and negative in Ethiopia and Nigeria. In other words, and contrary to the potential for transactions to enhance efficiency, in four of our countries rental activity either does not increase or is lower in more densely populated areas, possibly because of higher expropriation risk in these areas. This would be consistent with the fact that negative coefficients emerge in the two countries where the literature suggests expropriation risk in the context of urban expansion is highest, further testing of this hypothesis would be useful. Similarly, share of non-farm income remains insignificant throughout and, with the exception of Malawi, coefficients on distance to the next road are insignificant as well, pointing to at best a weak link between market access and growth of rental activities.

Regression results for land sale markets participation in Uganda and Niger using Eq. (1) but letting *R_ij_* be an indicator for land purchase during the last 5 years are reported in Table 7. Interestingly, in these two countries, the (marginally) more efficient and land-poor but not asset-poor purchased land and coefficients on geographic variables remain largely insignificant.

## 5 Conclusion and policy implications

The above analysis suggests that land markets are more active and have potential to contribute to structural transformation in Africa than commonly assumed. Analytically, this points towards a need to replace traditional views on African agriculture with a more differentiated and empirically grounded view. Consistent micro-data, together with an understanding of the institutional context, can help to identify ways in which this potential can be utilized. In the cases considered here, land market performance seems to be lower where implicit or explicit restrictions on land rental exist, where perceived threats of uncompensated expropriation reduce subjective tenure insecurity, and where policies to document existing land rights exist but are not implemented or implemented in an ad hoc manner or in a way that leaves out specific groups of land holders, in particular women, or is unaffordable to them. Further study to explore these possible links in more detail, possibly harnessing within-country variation, would be of interest.

Methodologically, our analysis points towards a number of areas where improvements in household questionnaire design and associated data collection protocols on land could make data even more useful. Key areas in this respect are (i) a more consistent recording of the history of land acquisition (to approximate land purchases); (ii) identifying rights held by individuals, including identification of plot manager and owner and possibly linking to land acquisition or inheritance history; (iii) providing an explanation for the discrepancies between land rented in and rented out (e.g. by asking questions to trace landlords for rented in or tenants for rented out parcels); (iv) training enumerators to distinguish different types of formal and informal documents (including maps) that may have been obtained to document ownership; (v) including proxy variables -e.g. likelihood of still owning the plot in 5 years or subjective risk of dispossession with or without compensation- of tenure insecurity; and (vi) cross-checking actual or hypothetical land prices to ensure their realism. This should go hand in hand with efforts to improving capacity for analysis of such data. Further opportunities to understand structural transformation in Africa and beyond can be harnessed by linking household data with remotely sensed imagery and administrative data.

From a policy perspective, our results imply a need to complement the focus on land administration and certification of rights with a broader perspective that takes into account restrictions on land transactions as well as the ways in which the growing demand for land from non-agricultural uses, especially urbanization, industry, and infrastructure, and from investors generally, is dealt with. To the extent that the recommended simple improvements in terms of questionnaire design are implemented, ready availability of high quality micro-level data of the type analyzed here will be essential to provide the information to do so.

## Figures and Tables

**Table A1:** Determinants of land rental market participation.

	Ethiopia	Malawi	Niger	Nigeria	Tanzania	Uganda
	Rent out	Rent in	Rent in	Rent in	Rent in	Rent in
Owned area (ha)		−0.141⁎⁎⁎	−0.010⁎⁎⁎	−0.129⁎⁎⁎	−0.022⁎⁎⁎	−0.108⁎⁎⁎
		(0.007)	(0.003)	(0.030)	(0.003)	(0.015)

# of <14 years old	0.007	0.004⁎⁎⁎	0.009⁎⁎⁎	0.011⁎⁎	0.004	0.004
	(0.004)	(0.001)	(0.003)	(0.006)	(0.003)	(0.004)

# of 14–60 years old	−0.011⁎	0.011⁎⁎⁎	0.010⁎⁎⁎	0.000	0.004	0.014⁎⁎
	(0.006)	(0.002)	(0.004)	(0.005)	(0.003)	(0.006)

# of >60 years old	0.016	−0.007	0.018	−0.003	−0.025⁎⁎	−0.036⁎⁎
	(0.012)	(0.006)	(0.013)	(0.023)	(0.011)	(0.018)

Age of head/10	0.011⁎⁎	−0.002	−0.008	0.003	−0.003	−0.001
	(0.005)	(0.002)	(0.005)	(0.009)	(0.004)	(0.007)

Illiterate head	0.010	−0.007	0.006	−0.010	0.001	−0.037
	(0.037)	(0.006)	(0.023)	(0.029)	(0.015)	(0.027)

Head has primary	0.013	−0.001	−0.002	0.020	0.002	0.007
	(0.042)	(0.005)	(0.023)	(0.023)	(0.013)	(0.021)

Female head	0.086⁎⁎⁎	−0.012⁎⁎⁎	−0.036⁎⁎	−0.051⁎⁎	−0.002	−0.007
	(0.023)	(0.004)	(0.016)	(0.020)	(0.010)	(0.016)

Mbr migrated in		0.024⁎⁎⁎			0.026	0.042⁎
		(0.008)			(0.018)	(0.024)

Mbr migrated out			0.032⁎			0.010
			(0.017)			(0.031)

Value of durable goods/1000		0.010⁎⁎⁎	0.003⁎	0.000		0.000
		(0.001)	(0.002)	(0.001)		(0.000)

Household consumption/1000	−0.037	0.005⁎⁎	−0.009⁎	−0.001	0.002	−0.002
	(0.026)	(0.002)	(0.005)	(0.005)	(0.003)	(0.007)

Ln output value/ha		0.050⁎⁎⁎	0.026⁎	0.023	−0.013	0.040
		(0.010)	(0.014)	(0.037)	(0.015)	(0.030)

Ln output value/ha squared		−0.006⁎⁎⁎	−0.003⁎⁎	−0.005	0.001	−0.003
		(0.001)	(0.002)	(0.003)	(0.001)	(0.003)

Rural pop. density (1000/sq km)	−0.048	0.066⁎⁎⁎	0.299⁎⁎⁎	−0.215⁎⁎⁎	−0.031	−0.066
	(0.071)	(0.024)	(0.116)	(0.080)	(0.060)	(0.058)

Share of non age wage income (EA)	−0.020	−0.023	0.093	0.003	−0.037	0.100
	(0.368)	(0.021)	(0.091)	(0.074)	(0.048)	(0.091)

Dist. to the nearest major road	−0.517	−0.443⁎⁎	−0.247	0.368	−0.075	1.175
	(0.451)	(0.218)	(0.621)	(0.617)	(0.202)	(1.647)

Rural area		0.013⁎	0.025	−0.025	−0.011	0.012
		(0.007)	(0.018)	(0.039)	(0.016)	(0.031)

Observations	1686	8390	1899	903	1698	1913
Pseudo R-squared	0.131	0.267	0.088	0.195	0.114	0.161

*Note:* Robust standard errors clustered by EA in parentheses.

⁎⁎⁎ p < 0.01.

⁎⁎ p < 0.05.

⁎ p < 0.1.

**Table 1:** Descriptive statistics at household level.

	Ethiopia 2011/12	Malawi 2010/11	Niger 2010/11	Nigeria 2010/11	Tanzania 2010/11	Uganda 2010/11
*Basic household characteristics*						
Share of landless	0.02	0.05	0.02	0.08	0.02	0.05
Share agric. cultivators	0.99	1.00	0.99	1.00	0.94	0.98
Household size	5.15	4.75	6.82	6.02	5.69	6.04
Adults (14–60)	2.57	2.36	3.06	3.09	2.96	3.18
Children (<14)	1.52	2.15	3.52	2.58	2.35	2.86
Old people (>60)	0.24	0.24	0.24	0.35	0.37	0.33
Head’s age	44.58	43.04	44.90	50.60	48.88	47.35
Head female	0.21	0.25	0.08	0.12	0.23	0.30
Head never attended school	0.66	0.23	0.50	0.40	0.29	0.16
Head has primary educ.	0.27	0.57	0.44	0.29	0.61	0.57
Head has more than primary educ.	0.04	0.19	0.06	0.30	0.10	0.22
Value of agricultural assets (USD)		323.61	483.73	340.46	415.95	24.49
Value of animals (USD)	499.70	85.98	489.79	185.67	266.45	369.41
Value of durable goods (USD)		1718.56	4402.32	1301.66		2948.10
Consumption (USD)	465.85	1452.10	2547.66	2529.20	1949.23	1093.05
Has members who migrated in for work/land		0.13			0.09	0.17
Has members who migrated out for work/land			0.21			0.08
Share of rural households	0.94	0.91	0.85	0.87	0.84	0.88

*Income and its sources*						
Gross household income (USD)		663.50	1395.64	1307.00	1159.81	1201.75
Gross income per capita (USD)		205.56	309.27	323.27	299.23	268.77
Share from crops		0.51	0.44	0.70	0.51	0.46
Share from livestock		0.10	0.17	0.09	0.10	0.19
Share from age wage		0.15	0.02	0.01	0.05	0.06
Share from non-age wage		0.09	0.06	0.08	0.11	0.10
Share from self-employment		0.06	0.24	0.11	0.17	0.13
Share from transfer		0.08	0.07	0.01	0.07	0.06

*Details of agricultural production*						
Gross inc. from crops (USD/ha)		515.57	267.09	2233.80	509.88	545.39
Net inc. from crops (USD/ha)		164.11	226.13	1196.67	289.16	279.06
Gross inc. from crops (USD)		266.48	517.01	543.06	434.02	406.27
Gross inc. per capita from crops (USD)		84.79	117.51	142.57	116.90	92.78
# of days by family members	126.82	74.28	151.07	108.76	183.03	234.95
Imputed return to family labor/day		2.21	1.50	11.00	1.58	1.19
Value of hired labor (USD)	20.47	3.56	41.93	27.05	17.79	30.35
Value of fertilizer (USD)		71.85	45.61	53.90	21.85	8.57

*Land ownership*						
No. of plots owned	3.02	1.74	2.74	1.75	2.22	1.88
Has any type of document	0.38	0.01	0.11		0.14	0.19
Area share with any document	0.48	0.01	0.07	0.00	0.13	0.17
Area share with formal document	0.48	0.01	0.04	0.00	0.03	0.12
Owned area ha	1.05	0.68	5.17	0.73	2.50	1.44
o/w granted by leaders/ext. family	0.48	0.15	0.00	0.78		0.00
o/w inherited	0.40	0.78	0.70	0.00		0.62
o/w purchased	0.00	0.06	0.06	0.08		0.26
o/w acquired by other means	0.13	0.03	0.24	0.14		0.13
Operated area ha	1.16	0.72	5.17	0.79	2.12	1.40
Owned area/adult (14–60)	0.44	0.32	1.93	0.28	0.90	0.53
Operated area/adult (14–60)	0.49	0.33	1.92	0.30	0.78	0.51
Male manager	0.80	0.71	0.53	0.81	0.27	0.09
Joint manager			0.36	0.01	0.45	0.52
Female manager	0.19	0.29	0.10	0.18	0.24	0.38
Male owner	0.44	0.35	0.58	0.75	0.40	0.25
Joint owner	0.25	0.17	0.26		0.31	0.46
Female owner	0.18	0.45	0.09	0.05	0.22	0.28

*Geographic variables*						
Population density (persons/sq. km)	142.32	175.28	49.07	207.37	64.99	268.53
Distance to nearest major road (km)	17.38	9.46	12.92	17.20	22.09	8.06
Light intensity	20.64	126.83	102.56	210.33	123.38	65.58
Urban gravity	7.04	137.15	41.36	116.35	60.40	53.22
No. of households	3099	10,123	2263	3029	2625	2129

**Table 2:** Details on land market participation at household level.

	Ethiopia	Malawi	Niger	Nigeria	Tanzania	Uganda
Autarkic (%)	73.99	89.65	91.12	89.04	92.69	80.55
Rent in (%)	20.81	10.06	7.29	10.80	6.17	18.65
If yes, mean area rented in	0.63	0.49	1.87	0.71	0.82	0.52
If yes, area rented in of operated area (%)		74.69	48.14	87.54	72.64	57.11
Rent out (%)	5.20	0.30	1.59	0.17	1.14	0.80
Total area rented in (with weights)	1,516,979	130,155	262,079	1,069,316	400,091	492,903
Total area rented out (with weights)	206,339	5092	80,486	15,404	197,619	27,824
Total area rented out/Total area rented in (%)	13.60	3.91	30.71	1.44	49.39	5.64
Gini of owned area	0.50	0.40	0.45	0.53	0.59	0.49
Gini of operated area	0.50	0.39	0.45	0.53	0.59	0.48
Purchased any during last 5 years (%)			3.58			9.63
If yes, mean area purchased			2.13			0.57
If yes, area purchased of owned area (%)			53.44			56.68

**Table 3:** Descriptive statistics by quartile of land ownership and productivity distribution.

	Ethiopia	Malawi	Niger	Nigeria	Tanzania	Uganda
Panel A: Land ownership distribution (ha)						
*Owned area*						
Mean	1.05	0.68	5.17	0.73	2.50	1.44
Median	0.74	0.53	3.86	0.44	1.25	0.95
Bottom quartile	0.12	0.15	0.93	0.03	0.28	0.20
2nd quartile	0.51	0.42	2.81	0.25	0.87	0.68
3rd quartile	1.05	0.69	5.35	0.74	1.88	1.36
Top quartile	2.53	1.48	11.60	1.88	6.97	3.54
Top quartile/bottom quartile	21.08	9.87	12.47	62.67	24.89	17.70
3rd quartile/2nd quartile	2.06	1.64	1.90	2.96	2.16	2.00
Skewness	2.67	3.68	1.88	1.91	5.69	2.67

*Owned area/adult (14–60)*						
Mean	0.44	0.32	1.93	0.28	0.90	0.53
Median	0.31	0.24	1.41	0.16	0.49	0.34
Bottom quartile	0.06	0.08	0.42	0.02	0.13	0.09
2nd quartile	0.25	0.22	1.20	0.11	0.38	0.30
3rd quartile	0.46	0.34	2.19	0.30	0.78	0.55
Top quartile	0.97	0.62	3.88	0.67	2.28	1.18
Top quartile/bottom quartile	16.17	7.75	9.24	33.50	17.54	13.11
3rd quartile/2nd quartile	1.84	1.55	1.83	2.73	2.05	1.83
Skewness	3.55	3.68	2.50	2.44	6.30	6.52

Panel B: Productivity distribution (USD)						
Mean		164.11	226.13	1196.67	289.16	279.06
Median		96.25	73.68	316.42	121.92	147.24
Bottom quartile		3.13	5.60	7.37	18.40	14.55
2nd quartile		58.78	47.67	172.63	81.14	91.84
3rd quartile		150.51	107.98	594.63	185.72	229.16
3rd quartile/2nd quartile		0.39	0.44	0.29	0.44	0.40
Top quartile		444.01	744.19	4012.04	871.98	781.67

Panel C: Productivity distribution (USD) by cultivated area						
Bottom quartile		244.68	625.88	2947.21	781.98	596.59
2nd quartile		159.80	137.62	1099.28	239.43	276.37
3rd quartile		145.11	99.03	500.72	171.10	178.28
Top quartile		106.60	52.99	267.21	87.17	86.33
Top quartile/bottom quartile		0.44	0.08	0.09	0.11	0.14
3rd quartile/2nd quartile		0.91	0.72	0.46	0.71	0.65
Skewness		7.74	24.06	13.19	29.61	8.77

Panel D: Operational land distribution (ha)						
*Operated area*						
Mean	1.16	0.72	5.17	0.79	2.12	1.40
Median	0.83	0.57	3.90	0.52	1.02	0.94
Bottom quartile	0.24	0.29	1.12	0.29	0.35	0.44
2nd quartile	0.63	0.44	2.91	0.26	0.77	0.70
3rd quartile	1.14	0.71	5.32	0.75	1.62	1.28
Top quartile	2.62	1.45	11.34	1.88	5.74	3.19
Top quartile/bottom quartile	10.92	5.00	10.13	6.48	16.40	7.25
3rd quartile/2nd quartile	1.81	1.61	1.83	2.88	2.10	1.83
Skewness	2.41	3.77	1.86	1.87	6.18	2.27

*Operated area/adult (14–60)*						
Mean	0.49	0.33	1.92	0.30	0.78	0.51
Median	0.34	0.25	1.45	0.18	0.40	0.35
Bottom quartile	0.13	0.14	0.50	0.13	0.17	0.19
2nd quartile	0.31	0.23	1.24	0.11	0.34	0.30
3rd quartile	0.50	0.34	2.17	0.30	0.68	0.51
Top quartile	0.99	0.61	3.75	0.67	1.89	1.05
Top quartile/bottom quartile	7.62	4.36	7.50	5.15	11.12	5.53
3rd quartile/2nd quartile	1.61	1.48	1.75	2.73	2.00	1.70
Skewness	3.20	3.72	2.50	2.68	5.98	4.38

**Table 4:** Descriptive statistics by nature of land rental markets participation.

	Rent in			Rent in			Rent in		
	No	Yes		No	Yes		No	Yes	
	Ethiopia			Malawi			Niger		

*Basic household characteristics*									
Share of landless	0.00	0.09	⁎⁎⁎	0.00	0.54	⁎⁎⁎	0.00	0.25	⁎⁎⁎
Share agric. Cultivators	0.99	1.00	⁎⁎	1.00	1.00		0.99	1.00	
Household size	5.11	5.30	⁎⁎	4.70	5.22	⁎⁎⁎	6.76	7.55	⁎⁎⁎
Adults (14–60)	2.54	2.69	⁎⁎	2.31	2.79	⁎⁎⁎	3.05	3.28	⁎
Kids (<14)	1.50	1.59		2.14	2.31	⁎⁎⁎	3.48	4.04	⁎⁎⁎
Old people (>60)	0.26	0.15	⁎⁎⁎	0.25	0.12	⁎⁎⁎	0.24	0.23	
Female head	0.24	0.10	⁎⁎⁎	0.26	0.15	⁎⁎⁎	0.08	0.04	⁎⁎
Age of head	45.62	40.68	⁎⁎⁎	43.41	39.70	⁎⁎⁎	45.09	42.39	⁎⁎
Head never attended school	0.68	0.61	⁎⁎⁎	0.24	0.16	⁎⁎⁎	0.51	0.46	
Head has primary	0.26	0.32	⁎⁎⁎	0.58	0.49	⁎⁎⁎	0.44	0.47	
Head has more than primary	0.04	0.06	⁎⁎	0.18	0.35	⁎⁎⁎	0.05	0.07	
Consumption (USD)	490.01	369.62	⁎⁎⁎	1373.12	2158.5	⁎⁎⁎	2538.08	2669.5	
Has members who migrated in				0.12	0.30	⁎⁎⁎			
Has members who migrated out							0.20	0.32	⁎⁎⁎
Share of rural household	0.95	0.91	⁎⁎⁎	0.93	0.79	⁎⁎⁎	0.86	0.82	

*Income and its sources*									
Household gross income (USD)				614.03	1106.0	⁎⁎⁎	1381.78	1571.8	⁎
Share from crops				0.52	0.42	⁎⁎⁎	0.43	0.45	
Share from livestock				0.10	0.09	⁎⁎⁎	0.17	0.16	
Share from age wage				0.16	0.12	⁎⁎⁎	0.02	0.02	
Share from non-age wage				0.08	0.22	⁎⁎⁎	0.06	0.05	
Share from self employment				0.06	0.11	⁎⁎⁎	0.24	0.26	
Share from transfer				0.08	0.04	⁎⁎⁎	0.07	0.05	⁎⁎
Gross income per capita (USD)				194.98	300.23		306.60	343.17	
Gross inc. from crops (USD/ha)				519.77	478.00		268.86	244.78	
Net inc. from crops (USD/ha)				168.63	123.71	⁎⁎⁎	229.33	185.78	

*Land endowments*									
Owned area/adult (14–60)	0.45	0.40	⁎⁎	0.34	0.11	⁎⁎⁎	2.00	1.06	⁎⁎⁎
No. of plots owned	2.98	3.18	⁎	1.85	0.71	⁎⁎⁎	2.78	2.25	⁎⁎⁎
Owned area ha	1.06	1.02		0.73	0.26	⁎⁎⁎	5.32	3.28	⁎⁎⁎
Operated area ha	1.03	1.63	⁎⁎⁎	0.72	0.75		5.17	5.13	
Operated area/adult (14–60)	0.44	0.66	⁎⁎⁎	0.34	0.31	⁎⁎	1.94	1.72	
Has any type of document	0.36	0.48		0.01	0.03	⁎⁎⁎	0.11	0.13	
Male manager	0.77	0.90		0.70	0.79	⁎⁎⁎	0.53	0.58	
Joint manager							0.36	0.32	
Female manager	0.22	0.09		0.30	0.21	⁎⁎⁎	0.10	0.08	
Male owner	0.48	0.31		0.36	0.23	⁎⁎⁎	0.59	0.42	⁎⁎⁎
Joint owner	0.25	0.26		0.17	0.13	⁎	0.26	0.15	⁎⁎⁎
Female owner	0.22	0.08		0.47	0.20	⁎⁎⁎	0.09	0.07	

*Production*									
Gross inc. from crops (USD)				265.87	272.09		511.91	581.14	
Gross inc. per capita from crops (USD)				85.66	76.96		116.41	131.33	
# of days by family members	113.73	175.32	⁎⁎⁎	74.79	69.73	⁎⁎	149.63	169.30	
Value of hired labor (USD)	20.23	21.36		3.21	6.70	⁎⁎⁎	41.55	46.67	
Value of fertilizer (USD)				69.52	93.00	⁎⁎⁎	42.92	79.45	⁎

*Assets*									
Value of agricultural assets (USD)				322.89	330.03		479.98	531.51	⁎⁎
Value of animals (USD)	510.00	460.54	⁎	86.90	77.77	⁎	490.27	483.62	
Value of durable goods (USD)				1604.3	2740.8	⁎⁎⁎	4344.84	5133.3	⁎⁎

*Geo variables*									
Share of non-age wage inc. (EA level)				0.09	0.16	⁎⁎⁎	0.06	0.07	⁎
Population density (persons/sq. km)	147.42	123.79		174.87	179.21		47.99	61.90	
Distance to the nearest major road (km)	17.35	17.52		9.72	7.15	⁎⁎⁎	13.25	8.67	⁎⁎⁎
Light intensity	22.17	14.83		110.02	277.15	⁎⁎⁎	101.99	109.80	
Urban gravity	5.29	13.69		132.28	180.71	⁎⁎⁎	41.78	36.00	
No. of households	2454	645		9105	1018		2098	165	

	Nigeria			Tanzania			Uganda		

*Basic household characteristics*									
Share of landless	0.00	0.74	⁎⁎⁎	0.00	0.35	⁎⁎⁎	0.00	0.24	⁎⁎⁎
Share agric. Cultivators	1.00	1.00		0.93	0.99	⁎⁎⁎	0.98	1.00	⁎⁎⁎
Household size	6.02	6.09		5.68	5.75		6.08	5.82	
Adults (14–60)	3.09	3.10		2.96	3.00		3.21	3.03	⁎
Kids (<14)	2.57	2.71		2.34	2.59		2.87	2.80	
Old people (>60)	0.36	0.27	⁎⁎	0.39	0.15	⁎⁎⁎	0.36	0.21	⁎⁎⁎
Female head	0.12	0.14		0.24	0.21		0.30	0.31	
Age of head	50.79	49.11	⁎	49.25	43.20	⁎⁎⁎	48.04	44.33	⁎⁎⁎
Head never attended school	0.41	0.36	⁎	0.29	0.21	⁎⁎	0.17	0.12	⁎⁎⁎
Head has primary	0.29	0.30		0.60	0.69	⁎⁎	0.57	0.60	
Head has more than primary	0.29	0.33		0.10	0.09		0.22	0.22	
Consumption (USD)	2500.43	2762.80	⁎⁎	1948.62	1958.5		1087.21	1119.0	
Has members who migrated in				0.09	0.14	⁎	0.16	0.22	⁎⁎⁎
Has members who migrated out							0.08	0.10	
Share of rural household	0.88	0.80	⁎⁎⁎	0.85	0.78	⁎⁎⁎	0.89	0.83	⁎⁎⁎

*Income and its sources*									
Mean household gross income (USD)	1301.77	1350.2		1149.79	1312.1		1147.17	1439.9	⁎⁎⁎
Share from crops	0.70	0.69		0.51	0.49		0.45	0.48	⁎
Share from livestock	0.10	0.07	⁎⁎	0.10	0.08		0.20	0.14	⁎⁎⁎
Share from age wage	0.01	0.01		0.05	0.05		0.06	0.07	
Share from non-age wage	0.08	0.11	⁎⁎	0.11	0.09		0.09	0.14	⁎⁎⁎
Share from self employment	0.11	0.12		0.17	0.25	⁎⁎⁎	0.14	0.12	
Share from transfer	0.01	0.00		0.07	0.04	⁎⁎	0.06	0.06	
Gross income per capita (USD)	320.85	343.32		297.47	326.02		250.20	349.81	
Gross inc. from crops (USD/ha)	2286.8	1796.6	⁎	494.09	735.14	⁎	503.20	725.39	⁎⁎⁎
Net inc. from crops (USD/ha)	1204.2	1134.20		284.43	356.55		256.67	374.62	⁎⁎⁎

*Land endowments*									
Owned area/adult (14–60)	0.30	0.05	⁎⁎⁎	0.94	0.35	⁎⁎⁎	0.61	0.20	⁎⁎⁎
No. of plots owned	1.90	0.49	⁎⁎⁎	2.28	1.27	⁎⁎⁎	2.04	1.15	⁎⁎⁎
Owned area ha	0.79	0.19	⁎⁎⁎	2.60	0.98	⁎⁎⁎	1.64	0.57	⁎⁎⁎
Operated area ha	0.78	0.89	⁎⁎	2.15	1.59	⁎	1.48	1.07	⁎⁎⁎
Operated area/adult (14–60)	0.30	0.34	⁎⁎	0.79	0.59	⁎	0.54	0.38	⁎⁎⁎
Has any type of document				0.15	0.09	⁎⁎	0.17	0.26	⁎⁎⁎
Share of plots with gender of manager	0.97	0.96		0.93	0.93		0.95	0.96	
Male manager	0.82	0.73	⁎⁎⁎	0.27	0.32		0.09	0.10	
Joint manager	0.01	0.00		0.45	0.42		0.54	0.45	⁎⁎⁎
Female manager	0.17	0.26	⁎⁎⁎	0.24	0.21		0.37	0.44	⁎⁎⁎
Share of plots with gender of owner	0.07	0.05		0.85	0.31		0.98	0.98	
Male owner	0.77	0.62	⁎	0.40	0.27	⁎⁎⁎	0.26	0.20	⁎⁎⁎
Joint owner				0.32	0.15	⁎⁎⁎	0.46	0.47	
Female owner	0.05	0.05		0.23	0.12	⁎⁎	0.28	0.32	⁎

*Production*									
Gross inc. from crops (USD)	542.02	552.21		428.99	506.18	⁎⁎	387.61	485.66	⁎⁎⁎
Gross inc. per capita from crops (USD)	142.64	141.97		116.33	125.07		87.98	113.23	
# of days by family members	106.76	125.31		185.16	152.63	⁎⁎	232.96	243.43	
Value of days by hired labor (USD)	26.94	28.03		17.25	25.45	⁎	32.53	21.10	⁎⁎⁎
Value of used fertilizer (USD)	56.44	31.73	⁎⁎	21.24	30.58		8.10	10.59	

*Assets*									
Value of agricultural assets (USD)	145.45	1951.8	⁎⁎⁎	409.93	507.48		26.40	16.14	
Value of animals (USD)	194.94	109.10	⁎⁎⁎	268.50	235.34		403.90	218.93	
Value of durable goods (USD)	1295.6	1351.9					2839.35	3422.6	

*Geo variables*									
Share of non-age wage income (EA level)	0.08	0.10	⁎⁎	0.11	0.09		0.10	0.12	⁎⁎⁎
Population density (persons/sq. km)	212.44	166.46		65.06	63.98		266.06	279.40	
Distance to the nearest major road (km)	17.30	16.36		22.33	18.35	⁎⁎	8.20	7.41	⁎
Light intensity	200.44	292.02	⁎⁎⁎	123.47	122.07		52.63	122.38	⁎⁎⁎
Urban gravity	110.34	165.98	⁎⁎⁎	61.56	42.73		38.60	117.32	⁎⁎⁎
No. of households	2702	327		2463	162		1732	397	

*Note:* Tests for differences of means between lessees and non lessees are reported with

⁎ Significant at 10%.

⁎⁎ Significant at 5%.

⁎⁎⁎ Significant at 1%.

**Table 5:** Descriptive statistics by land sale market participation.

	Niger			Uganda		
	Autarky or sold	Purchased		Autarky or sold	Purchased	
*Basic household characteristics*						
Share of landless	0.02	0.00		0.05	0.00	⁎⁎⁎
Share agric. Cultivators	0.99	1.00		0.98	1.00	⁎⁎
Household size	6.79	7.80	⁎⁎⁎	6.00	6.39	⁎
Adults (14–60)	3.05	3.41	⁎	3.19	3.04	
Kids (<14)	3.49	4.25	⁎⁎⁎	2.81	3.35	⁎⁎⁎
Old people (>60)	0.24	0.15	⁎	0.35	0.13	⁎⁎⁎
Female head	0.08	0.02	⁎	0.31	0.19	⁎⁎⁎
Age of head	44.98	42.65		48.04	40.86	⁎⁎⁎
Head never attended school	0.51	0.38	⁎⁎	0.17	0.11	⁎⁎
Head has primary	0.44	0.54	⁎	0.58	0.52	⁎
Head has more than primary	0.05	0.07		0.21	0.29	⁎⁎⁎
Consumption (USD)	2504.52	3709.88	⁎⁎⁎	1067.99	1332.51	⁎⁎⁎
Has members who migrated in				0.16	0.28	⁎⁎⁎
Has members who migrated out	0.20	0.27		0.08	0.10	
Share of rural household	0.86	0.79	⁎	0.88	0.88	

*Income and its sources*						
Mean household gross income (USD)	1381.66	1772.24	⁎⁎⁎	1155.10	1639.64	⁎⁎⁎
Share from crops	0.44	0.44		0.46	0.44	
Share from livestock	0.17	0.21	⁎	0.19	0.17	
Share from age wage	0.02	0.00	⁎⁎	0.06	0.06	
Share from non-age wage	0.06	0.05		0.10	0.14	⁎⁎
Share from self employment	0.24	0.24		0.13	0.17	⁎⁎
Share from transfer	0.07	0.06		0.06	0.03	⁎⁎⁎
Gross income per capita (USD)	308.60	327.16		255.84	390.18	
Gross inc. from crops (USD/ha)	269.25	209.32		528.80	697.97	⁎
Net inc. from crops (USD/ha)	230.11	119.68		268.19	379.12	⁎⁎⁎

*Land endowments*						
Owned area/adult (14–60) five years ago	1.94	1.09	⁎⁎⁎	0.65	0.30	⁎⁎⁎
Owned area/adult (14–60)	1.93	1.96		0.54	0.47	
No. of plots owned five years ago	2.20	1.68	⁎⁎	1.69	1.26	⁎⁎⁎
No. of plots owned	2.70	3.75	⁎⁎⁎	1.80	2.58	⁎⁎⁎
Owned area ha five years ago	4.43	2.72	⁎⁎⁎	1.38	0.70	⁎⁎⁎
Owned area ha	5.15	5.62		1.46	1.31	
Operated area ha	5.16	5.33		1.41	1.35	
Operated area/adult (14–60)	1.93	1.81		0.51	0.49	
Has any type of document	0.09	0.46	⁎⁎⁎	0.16	0.43	⁎⁎⁎
Share of plots with gender of manager	0.96	0.97		0.95	0.97	
Male manager	0.53	0.68	⁎⁎⁎	0.10	0.06	
Joint manager	0.36	0.24	⁎⁎	0.51	0.58	⁎
Female manager	0.10	0.08		0.39	0.35	
Share of plots with gender of owner	0.78	0.90		0.98	0.99	
Male owner	0.58	0.73	⁎⁎⁎	0.25	0.31	⁎⁎
Joint owner	0.26	0.11	⁎⁎⁎	0.45	0.52	⁎⁎
Female owner	0.09	0.07		0.30	0.16	⁎⁎⁎

*Production*						
Gross inc. from crops (USD)	513.49	611.57		394.94	510.19	⁎⁎⁎
Gross inc. per capita from crops (USD)	117.60	115.12		89.96	118.71	
# of days by family members	148.73	213.67	⁎⁎⁎	233.37	249.44	
Value of days by hired labor (USD)	39.17	116.05	⁎⁎⁎	28.65	45.97	⁎⁎⁎
Value of used fertilizer (USD)	44.90	64.66		6.89	24.03	⁎⁎⁎

*Assets*						
Value of agricultural assets (USD)	481.79	536.08	⁎	24.64	23.03	
Value of animals (USD)	482.48	686.53	⁎⁎	366.97	392.27	
Value of durable goods (USD)	4348.33	5856.95	⁎⁎⁎	2607.45	6145.21	

*Geo variables*						
Share of non-age wage income (EA level)	0.06	0.07		0.10	0.10	
Population density (persons/sq. km)	48.43	65.09		265.21	300.51	
Distance to the nearest major road (km)	12.92	12.90		8.08	7.87	
Light intensity	101.14	140.93		61.11	107.71	⁎
Urban gravity	41.58	35.30		52.07	64.05	
No. of households	2182	81		1924	205	

*Note:* Tests of differences of means between lessees and non lessees are reported with

⁎ Significant at 10%.

⁎⁎ Significant at 5%.

⁎⁎⁎ Significant at 1%.

**Table 6:** Determinants of land rental market participation.

	Ethiopia	Malawi	Niger	Nigeria	Tanzania	Uganda
	Rent in	Rent in	Rent in	Rent in	Rent in	Rent in
Owned area (ha)	−0.038⁎⁎⁎	−0.142⁎⁎⁎	−0.010⁎⁎⁎	−0.117⁎⁎⁎	−0.019⁎⁎⁎	−0.108⁎⁎⁎
	(0.014)	(0.007)	(0.002)	(0.030)	(0.003)	(0.014)

# of <14 years old	0.031⁎⁎⁎	0.003⁎⁎⁎	0.008⁎⁎⁎	0.011⁎⁎	0.004⁎	0.003
	(0.010)	(0.001)	(0.003)	(0.006)	(0.002)	(0.004)

# of 14–60 years old	0.025⁎⁎	0.011⁎⁎⁎	0.010⁎⁎⁎	0.002	0.004	0.014⁎⁎
	(0.010)	(0.002)	(0.004)	(0.006)	(0.003)	(0.006)

# of >60 years old	−0.003	−0.006	0.017	−0.004	−0.024⁎⁎	−0.034⁎
	(0.031)	(0.006)	(0.013)	(0.023)	(0.010)	(0.017)

Age of head/10	−0.034⁎⁎⁎	−0.002	−0.008	0.001	−0.003	−0.002
	(0.012)	(0.002)	(0.005)	(0.009)	(0.003)	(0.007)

Illiterate head	−0.020	−0.004	0.004	−0.000	0.002	−0.040
	(0.066)	(0.006)	(0.023)	(0.031)	(0.014)	(0.026)

Head has primary	0.002	−0.003	−0.003	0.010	0.004	0.009
	(0.064)	(0.005)	(0.022)	(0.025)	(0.012)	(0.021)

Female head	−0.157⁎⁎⁎	−0.011⁎⁎⁎	−0.036⁎⁎	−0.039⁎	−0.001	−0.009
	(0.028)	(0.004)	(0.016)	(0.021)	(0.009)	(0.016)

Mbr migrated in		0.029⁎⁎⁎			0.019	0.045⁎
		(0.008)			(0.016)	(0.024)

Mbr migrated out			0.032⁎			0.000
			(0.017)			(0.029)

Durable good val./1000		0.010⁎⁎⁎	0.003	−0.000		0.000
		(0.001)	(0.002)	(0.002)		(0.000)

HH cons./1000	−0.126⁎⁎⁎	0.005⁎⁎	−0.010⁎	−0.004	0.001	−0.000
	(0.043)	(0.002)	(0.005)	(0.005)	(0.003)	(0.006)

Rural pop. Dens (1000/km^2^)	−0.555⁎⁎⁎	0.071⁎⁎⁎	0.339⁎⁎⁎	−0.223⁎⁎	−0.047	−0.047
	(0.168)	(0.026)	(0.115)	(0.088)	(0.055)	(0.056)

Share non age wage inc. (EA)	−0.573	−0.022	0.097	0.033	−0.038	0.063
	(1.059)	(0.019)	(0.086)	(0.074)	(0.043)	(0.088)

Dist. to the nearest road	0.460	−0.484⁎⁎	−0.279	0.370	−0.112	1.207
	(1.390)	(0.223)	(0.605)	(0.749)	(0.190)	(1.648)

Rural dummy		0.013⁎	0.027	−0.041	−0.012	0.014
		(0.007)	(0.017)	(0.047)	(0.015)	(0.030)

Observations	1514	9166	1946	988	1866	1961
Pseudo R-squared	0.093	0.256	0.085	0.165	0.106	0.156

*Note:* Robust standard errors clustered by EA in parentheses.

⁎⁎⁎ p < 0.01.

⁎⁎ p < 0.05.

⁎ p < 0.1.

**Table 7:** Determinants of land sale markets participation during last five years.

	Niger		Uganda	
Owned area five years ago (ha)	−0.003⁎⁎	−0.003⁎⁎	−0.032⁎⁎⁎	−0.033⁎⁎⁎
	(0.001)	(0.001)	(0.007)	(0.008)

# of < 14 years old five years ago	0.001	0.001	0.006⁎⁎	0.006⁎⁎
	(0.001)	(0.001)	(0.003)	(0.003)

# of 14–60 years old five years ago	0.001	0.000	−0.001	−0.001
	(0.004)	(0.004)	(0.005)	(0.005)

# of > 60 years old five years ago	−0.006	−0.005	−0.001	−0.001
	(0.010)	(0.010)	(0.007)	(0.007)

Age of head/10	−0.003	−0.003	−0.020⁎⁎⁎	−0.020⁎⁎⁎
	(0.003)	(0.003)	(0.004)	(0.005)

Illiterate head	0.014	0.016	−0.004	−0.003
	(0.014)	(0.014)	(0.019)	(0.020)

Head has primary	0.014	0.016	−0.030⁎⁎	−0.031⁎⁎
	(0.015)	(0.015)	(0.014)	(0.014)

Female head	−0.020⁎⁎	−0.020⁎⁎	−0.038⁎⁎⁎	−0.037⁎⁎⁎
	(0.008)	(0.008)	(0.012)	(0.012)

Has members who migrated in	0.001	0.001	0.000	0.000
	(0.001)	(0.001)	(0.000)	(0.000)

Has members who migrated out	0.008⁎⁎⁎	0.009⁎⁎⁎	0.007	0.007
	(0.002)	(0.002)	(0.004)	(0.005)

Value of durable goods/1000	0.001	0.001	0.000	0.000
	(0.001)	(0.001)	(0.000)	(0.000)

Household consumption/1000	0.008⁎⁎⁎	0.009⁎⁎⁎	0.009⁎⁎	0.009⁎
	(0.002)	(0.002)	(0.004)	(0.005)

ln(output value/operated area)		0.020⁎		0.020
		(0.012)		(0.020)

Quadratic ln(output value/operated area)		−0.002⁎		−0.002
		(0.001)		(0.002)

Rural population density (1000/sq km)	0.181⁎⁎	0.156⁎⁎	0.051	0.048
	(0.079)	(0.078)	(0.034)	(0.036)

Share of non age wage income (EA level)	−0.008	−0.012	−0.017	−0.038
	(0.044)	(0.046)	(0.052)	(0.053)

Distance to the nearest major road/1000	0.196	0.187	−0.719	−0.759
	(0.374)	(0.369)	(0.842)	(0.852)

Rural area	0.011	0.011	0.027⁎	0.022
	(0.011)	(0.011)	(0.015)	(0.016)

Observations	1946	1899	1961	1913
R-squared	0.104	0.111	0.111	0.112

*Note:* Robust standard errors are in brackets.

⁎ Significant at 10%;

⁎⁎ Significant at 5%;

⁎⁎⁎ Significant at 1%.

## Appendix A

See Table A1.

## Footnotes

1 Living Standards Measurement Study – Integrated Surveys on Agriculture implemented by the World Bank www.worldbank.org/lsms-isa.

2 Some African countries are quickly approaching or even surpassing levels of population density that are normally thought of to be more common in Asia. Rwanda, with 384 inhabitants/km^2^ overall and 526/km^2^ of agricultural land is a prominent example.

3 The fact that transactions are outlawed does of course not prevent them from happening but just drives them underground, depressing productivity and investment-effects (Deininger et al., 2013b). Well implemented household surveys are one of the few tools to reliably measure the incidence of such informal transactions.

4 In Ethiopia, the relevant information used is the plot holder’s ID, in Malawi it is the response (one person) to the question “who in the household makes the decisions concerning crops to be planted, input use and the timing of cropping activities on this plot” for one person in Malawi, in Niger it is the response to the question “what is the ID number of the person who currently works the parcel” for one person, and we identify the manager is joint if the whole household was reported rather than a personal ID, in Nigeria there are two questions on “who in the household manages this plot” (one person) and “write the ID codes of other household members who are decision makers on this plot” (up to four persons), and in Tanzania and Uganda, the manager is identified by the question of “who decided what to plant on this plot” (one person) or “who usually works on this parcel” (up to two persons), respectively. The extent to which information on the plot owner is provided is more limited. In different countries, the relevant person is identified in response to the following questions: “under whose name(s) is the certificate issued for this parcel” (up to two persons) in Ethiopia; “who in this household owns this plot” (up to two persons) in Malawi; “what is the ID number of the owner of this parcel” for one person with ‘the whole household’ as one of the codes in Niger; “who is the owner of this plot” (one person) in Nigeria; “who in the household owns this plot” (up to two persons) in Tanzania; and “who has the ownership/use rights to this parcel” (up to two persons) in Uganda.

5 In Tanzania, the total area cultivated by smallholders, using the weights from our survey, amounts to 6.026 million hectares, compared to 9.2 million hectares that are cultivated according to FAO statistics (Deininger and Byerlee, 2011).

6 In Ethiopia, output is estimated using crop-cutting of randomly selected crops/fields in each EA to be able to obtain statistically reliable estimates of output for different crops. This makes analysis of productivity at farm or household level impossible.

7 Income and productivity data are doubtful for Nigeria where there are some data issues with the level of gross income (Oseni et al., 2014).

8 We do not include presence of land documents in our regressions because comparison of answers for the same households across different survey rounds in Uganda suggests that, without proper training of enumerators, responses may contain significant measurement error (Ali et al., 2015a).

9 As this is an issue that will become of increasing relevance over time, finding ways to address it in household surveys will very relevant.

10 For the analysis of land rental markets to be conducted here, this implies that use of a maximum likelihood friction model that accounts for both sides of the market (Deininger et al., 2008c, Deininger et al., 2012) will be infeasible.

11 Obtaining landlord details from households leasing in as for example in Ethiopia (Deininger et al., 2013a), would be one way of addressing both concerns.

12 As the above discussion points towards considerable measurement error in the ‘leasing out’ variable, we group those who remain in autarky and rent out to compare with those who rent in.

13 In many countries, a desire to avoid taxes, together with an outdated valuation system, implies that prices for registered sales may be systematically under-declared, something that is often associated with weak governance.

14 Figures for land ownership are 2.72 ha vs. 4.43 ha for owned area, and 1.09 ha vs. 1.94 ha for owner area per adult in Niger and 0.70 ha vs. 1.38 ha for owned area, and 0.30 ha vs. 0.65 ha for owned area per adult in Uganda.

15 Ethiopia is the only country where the underlying household survey points to a share of lessors of more than 5%. As in 62% of cases, information on plot size for plots rented out is missing we can only run very basic regressions to assess determinants of market participation on both sides.

16 We run separate regressions with one geographic variable each time due to high correlations. Regression results on light intensity and urban gravity as well as separately for North and South Nigeria as suggested by Oseni et al. (2014) are available on request.

17 The survey for Ethiopia only asked for the quantity of assets, a measure that is not comparable with other countries and, without very strong assumptions on implicit asset values, is difficult to translate into monetary values. We thus have excluded it.
